# Data-Driven Modelling of Polyethylene Recycling under High-Temperature Extrusion

**DOI:** 10.3390/polym14040800

**Published:** 2022-02-18

**Authors:** Fanny Castéran, Karim Delage, Nicolas Hascoët, Amine Ammar, Francisco Chinesta, Philippe Cassagnau

**Affiliations:** 1 Centre National de la Recherche Scientifique, Ingénierie des Matériaux Polymères, Université Claude Bernard Lyon 1, 15 Boulevard André Latarjet, 69622 Villeurbanne, France; fanny.casteran@univ-lyon1.fr (F.C.); karim.delage@univ-lyon1.fr (K.D.); 2 ESI Group Chair@PIMM, Arts et Métiers Institute of Technology, 151 Boulevard de l’Hôpital, 75013 Paris, France; nicolas.hascoet@ensam.eu (N.H.); francisco.chinesta@ensam.eu (F.C.); 3 ESI Group Chair@LAMPA, Arts et Métiers Institute of Technology, 2 Boulevard du Ronceray, 49035 Angers, France; amine.ammar@ensam.eu

**Keywords:** polyethylene recycling, artificial engineering, polymer extrusion, machine learning

## Abstract

Two main problems are studied in this article. The first one is the use of the extrusion process for controlled thermo-mechanical degradation of polyethylene for recycling applications. The second is the data-based modelling of such reactive extrusion processes. Polyethylenes (high density polyethylene (HDPE) and ultra-high molecular weight polyethylene (UHMWPE)) were extruded in a corotating twin-screw extruder under high temperatures (350 °C < *T* < 420 °C) for various process conditions (flow rate and screw rotation speed). These process conditions involved a decrease in the molecular weight due to degradation reactions. A numerical method based on the Carreau-Yasuda model was developed to predict the rheological behaviour (variation of the viscosity versus shear rate) from the in-line measurement of the die pressure. The results were successfully compared to the viscosity measured from offline measurement assuming the Cox-Merz law. Weight average molecular weights were estimated from the resulting zero-shear rate viscosity. Furthermore, the linear viscoelastic behaviours (Frequency dependence of the complex shear modulus) were also used to predict the molecular weight distributions of final products by an inverse rheological method. Size exclusion chromatography (SEC) was performed on five samples, and the resulting molecular weight distributions were compared to the values obtained with the two aforementioned techniques. The values of weight average molecular weights were similar for the three techniques. The complete molecular weight distributions obtained by inverse rheology were similar to the SEC ones for extruded HDPE samples, but some inaccuracies were observed for extruded UHMWPE samples. The Ludovic^®^ (SC-Consultants, Saint-Etienne, France) corotating twin-screw extrusion simulation software was used as a classical process simulation. However, as the rheo-kinetic laws of this process were unknown, the software could not predict all the flow characteristics successfully. Finally, machine learning techniques, able to operate in the low-data limit, were tested to build predicting models of the process outputs and material characteristics. Support Vector Machine Regression (SVR) and sparsed Proper Generalized Decomposition (sPGD) techniques were chosen to predict the process outputs successfully. These methods were also applied to material characteristics data, and both were found to be effective in predicting molecular weights. More precisely, the sPGD gave better results than the SVR for the zero-shear viscosity prediction. Stochastic methods were also tested on some of the data and showed promising results.

## 1. Introduction

Considering the current situation of plastic consumption worldwide, the issue of end-of-life of polymer materials has become a significant problem. Polyethylene (PE) accounts for most plastic packaging and, consequently, plastic waste [1]. As PE is a thermoplastic, the most common method for its recycling is mechanical recycling, which involves reprocessing the materials [2,3,4]. These processes can induce the formation of radicals by homolytic cleavage of the polymers, inducing degradation, branching or even crosslinking of the materials leading to different final properties [5,6,7,8,9]. Consequently, to these properties changes, the applications of the mechanically recycled polymers have to be adapted [10,11,12]. Whereas the majority of recycled high and low-density polyethylenes (HDPE and LDPE) are however produced that way, Ultra High Molecular Weight Polyethylenes (UHMWPE), mostly used for high-performance applications due to their superior mechanical properties, are more difficult or impossible to process due to their high viscosity [13]. The other principal way of recycling polymers is by chemical recycling, which consists of a chemical transformation leading to new raw materials. Whereas polyethylene terephthalate (PET) can be depolymerized into dimethyl terephthalate and ethylene glycol by mathanolysis [14], no such reactions are possible for PE. The main way of PE chemical recycling is then pyrolysis, leading to smaller carbonated molecules, which can, in theory, be reinjected into the chemical industry [2,6,15,16,17,18,19,20,21].

**Figure 1:**
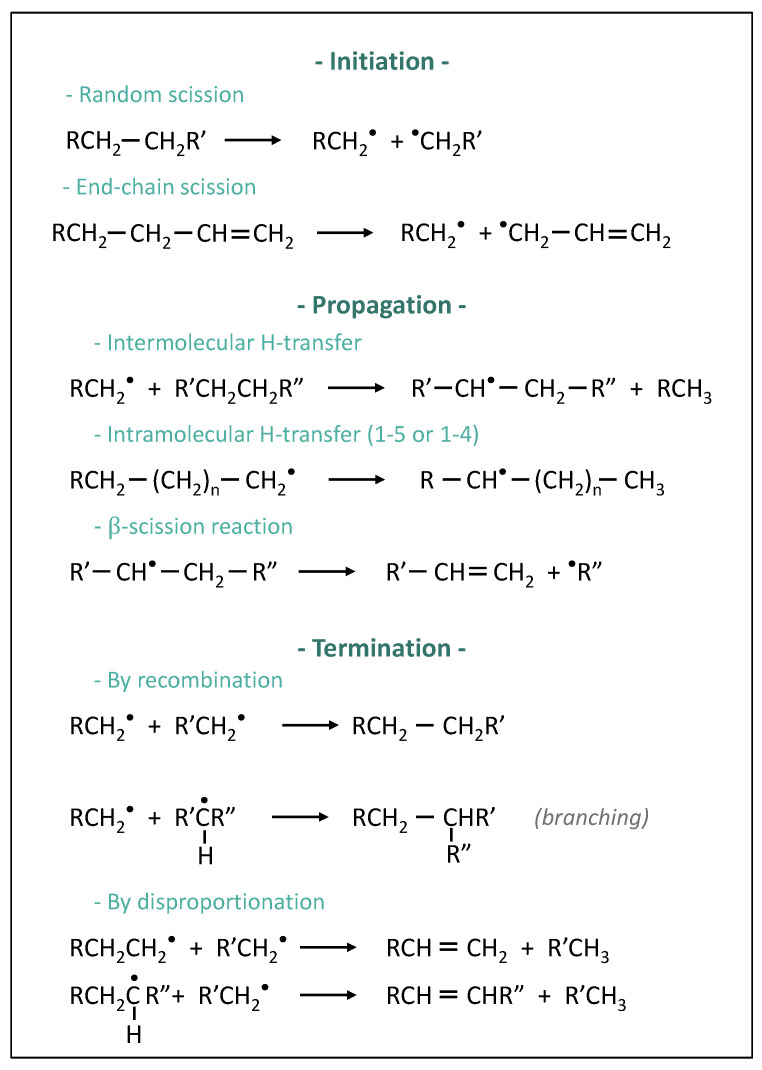
Simplified mechanisms of thermal degradation of polyethylene [23,24].

Whilst thermal degradation of polymers that include heteroatoms in their structure (PMMA, for instance) leads to simple products and mechanisms, PE degradation is more complex [22]. A simplification of PE degradation mechanisms is presented in Figure 1.

**Figure 2:**
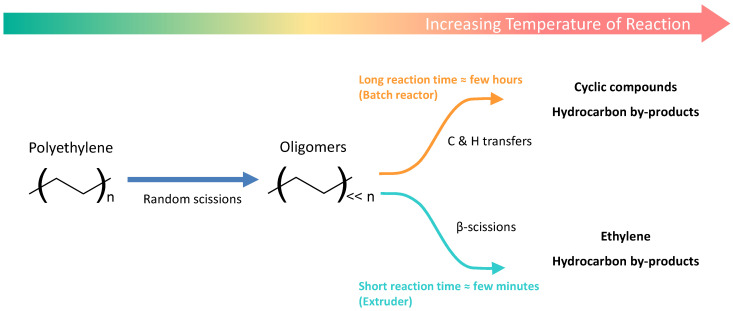
Representation of the different products of polyethylene degradation depending on temperature and residence time, inspired from Vollmer et al. [25].

As shown in this figure, different degradation mechanisms are possible for PE thermal degradation. The process conditions then define their probability. Whereas for polymers with a more complex structure, the end-chain scission mechanism would be preponderant, in the case of polyolefins, random scissions are more significant [24]. Therefore, the higher the molecular weight of the polyolefin, the more random scissions occur, resulting in a narrowing of the molecular weight distribution [25]. As schematized in Figure 2, the molecular weight decreases with the increase of the temperature of the reaction, leading first to oligomers and then to smaller molecules whose nature depends on propagation and termination mechanisms. A short reaction time at high temperatures would favour β-scissions, leading to the formation of a certain yield of the ethylene monomer. However, longer reaction times tend to favour the production of cyclic compounds due to intramolecular and intermolecular transfers, which are less easy to valorize afterwards [25].

The final products are then highly dependent on the processing conditions. Moreover, classic pyrolysis processes induce heat and mass transfer problems, leading to highly heterogeneous products without the possibility of controlling the degradation [25].

Other innovative ways of PE recycling were recently studied. Manas et al. [26,27], for instance, studied the recycling of PE crosslinked by irradiations by using it as a filler for virgin LDPE, Elmanowich et al. [28] studied the use of supercritical fluids for PE recycling, and recent promising studies are about the enzymatic degradation of polymers [29,30].

The present work aims at controlling the thermo-mechanical degradation of PE by carrying out a twin-screw extrusion process at high temperatures (320 < *T* °C < 420). Whereas polyethylene extrusion usually leads to branching and crosslinking, the extrusion thermal conditions in this work favour degradation mechanisms closer to pyrolysis conditions.

With the question of the process control comes the issue of its simulation. Extrusion simulation has been widely studied in the last decades. However, the complexity of the physical phenomena involved in the extrusion process involves either a long time and a significant computing power or a lot of hypotheses and simplifications. Furthermore, in this study, the viscoelastic properties of the materials evolve with their degradation. Due to the temperatures reached in the extruder, the materials are at the limit of pyrolysis. Moreover, the presence of oxygen involves additional chemical reactions due to high-temperature oxidation. Thus, the complexity of the degradation mechanisms increases the imprecision of the simulation.

On the other hand, machine learning does not need to understand these complex physics, only to have accurate experimental data to predict the results of new experiments. This approach often leads to faster computing and sometimes to more precise results, the imprecision coming from unknown phenomena or simplifications unavoidable in classical simulations that are not necessary with machine learning. Such methodologies were successfully employed in previous works on various reactive extrusion systems [31,32].

The following is a study of the degradation of HDPE and UHMWPE by twin-screw extrusion at high temperatures. Then, several methods will be applied and compared to determine the extruded polyethylenes’ final viscosities and molecular weights. Finally, the modelling of this process with the twin-screw extrusion Ludovic^®^ simulation software (SC-Consultants, Saint-Etienne, France) will be compared to the results obtained with Machine-Learning methodologies.

## 2. Experimental Section

### 2.1. Materials and Extrusion

HDPE (HDPE XRT70, TOTAL, Melt Flow Index (MFI: 190 °C/5 kg) = 0.7 g/10 min) and UHMWPE (GUR 4130, Celanese, Melt Flow Index (MFI: 190 °C/21.6 kg) < 0.1 g/10 min) were extruded in an intermeshing corotating twin-screw extruder ZSE 18 MAXX/HPe (Leistritz, Nuremberg, Germany) for various temperatures, screw rotation speeds and exit flow rates, reaching 29 different configurations. These processing conditions are summarized in Table 1.

**Figure 3:**
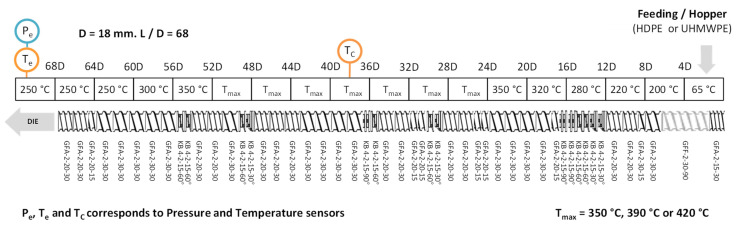
Scheme of the screw and temperature profiles used.

As the Polyethylene samples are highly viscous, and in order to have a maximum of possibilities concerning flow rate and rotation speed combinations, the screw profile was designed with quite a low shear and few restrictive elements. The temperature was increased progressively along with the barrel blocks and progressively decreased before the die for security issues and to limit the degradation at the die exit. The polyethylenes were introduced at the extruder entrance with a gravimetric feeder. The die had a 3 mm diameter. At the exit, the materials were cooled by air and then pelletized. The extruder configuration is described in Figure 3.

The details about the different extrusion configurations tested and the in-line measures are presented in the Appendix A of this article, in Table A1.

The torque, the Engine Power and the die pressure were measured for each experiment. Thermocouples placed around the centre of the extruder (*T_c_* at *L* = 38D) and in the die permitted to measure the melt temperature. However, it appears that the temperature measured is the one of the inner surface of the barrel and not precisely the bulk material temperature. Therefore, a manual thermocouple was used to measure it at the die exit of the extruder.

### 2.2. Characterizations

The rheological behaviour of the extruded and raw materials was studied using a DHR-2 (TA Instruments, New Castle, DE, USA), a stress-controlled rheometer. Frequency sweeps have been carried out at 190 °C from 100 to 0.01 rad·s^−1^ under nitrogen and with 1% deformation. The geometry used was 25 mm diameter parallel plates with a 1 mm gap.

The molecular weight distributions of five samples (two degraded HDPE, two degraded UHMWPE and raw HDPE XRT70) were measured using high-temperature steric exclusion chromatography (HT-SEC) using a Viscositek (Malvern Panalytical Ltd., Malvern, UK) device. The samples were previously dissolved in toluene at 100 °C for 30 min.

### 2.3. Theoretical Methodologies

#### 2.3.1. Determination of *Mw* from Viscoelastic Behaviour

The molecular weight distributions have been calculated using the TA instrument tool implemented in the Trios software (TA Instruments, New Castle, DE, USA). This tool, based on the double reptation theories [33,34], uses a model linking the molecular weight distribution to the relaxation modulus via the following relationship:

(1) $$G\left(t\right)=G_{N}^{0}\cdot {\left[\overset{\infty }{\underset{\ln\left(Me\right)}{\int }}F^{\frac{1}{\beta }}\;w\left(M\right)dln\left(M\right)\right]}^{\beta }$$

where G(t) is the linear viscoelastic relaxation modulus, $G_{N}^{0}$ is the plateau modulus, w(M)  is the molecular weight distribution, F(M,t) is the monodisperse relaxation function, and β takes the value 1 for simple reptation and 2 for double reptation. Several models exist to define the F function. In this study, as in most cases, a single exponential form described by Equation (2) is applied. Table 2 presents the definition of the constants used by the model and their values in this work, which correspond to classical values for polyethylene.

(2) $$F^{\frac{1}{2}}\left(M,t\right)=exp\left(\frac{-t}{2\lambda \left(M\right)}\right)\;\mathrm{with}\;\lambda \left(M\right)=K_{\lambda }\left(T\right)M^{\alpha };\;K_{\lambda }\left(T\right)=K_{\lambda }\left(T_{0}\right)\;exp\left(\frac{E_{a}}{RT}\right)$$

#### 2.3.2. Determination of *Mw* from Measured Die Pressure

**Figure 4:**
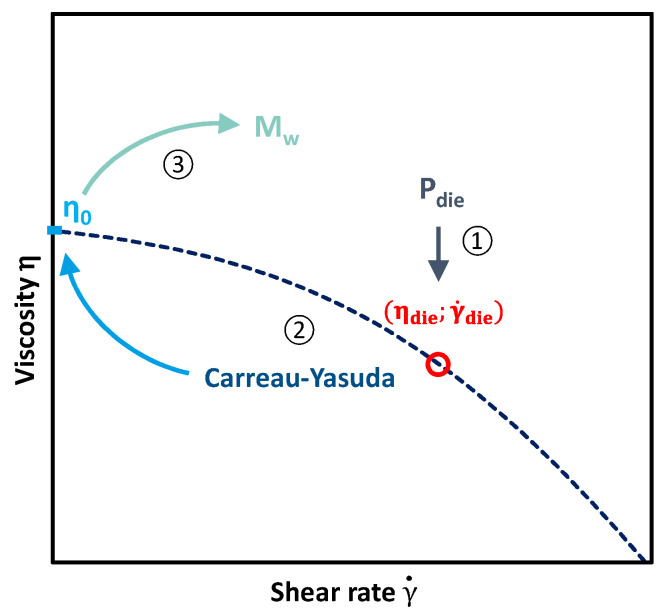
Scheme of the *Mw* determination from die pressure measurement.

As the pressure measured at the extruder die depends on the viscosity of the viscous polymer, which can be related to the molecular weight, it is then possible to estimate the viscosity and subsequently the molecular weight without the need for post-process characterizations. Figure 4 summarises the different steps required to measure *Mw* from the die pressure measurement.

The first step is to determine the value of the viscosity and the shear rate in the die. For this purpose, the die is considered a capillary rheometer. The experimentally measured temperatures and pressures are then approximated to those of the narrowest section of the die. As in a capillary rheometer, an apparent shear rate ${\dot{\gamma }}_{app}$ and an apparent viscosity $\;\eta_{app}$ are calculated as a function of the flow rate *Q*, the die cross-section and length *r* and *L* and the difference between die and outside drop pressure Δ*P* according to Equation (3).

(3) $${\dot{\gamma }}_{app}=\frac{4\;Q}{\pi \;r^{3}}\;\;\;\;;\;\;\;\;\eta_{app}=\frac{\Delta P\;r}{2\;L{\dot{\gamma }}_{app}}$$

These values are valid at the temperature of the die $\;T_{die}$, it is then necessary to calculate them at a reference temperature $\;T_{0}\;$ which is set at 190 °C in this study. This correction is then done assuming an Arrhenius dependence of the viscosity (Equation (4)).

(4) $$\eta_{app}\left(T_{die}\right)=a_{T}\times \eta_{app}\left(T_{0}\right)\;\;\;\;;\;\;\;\;a_{T}=exp\left(\frac{E_{a}}{R}\times \left(\frac{1}{T_{0}}-\frac{1}{T_{die}}\right)\right)$$

*E_a_* = activation energy;

*R* = ideal gases constant.

Then, the Rabinowitsch correction (Equation (5)) has to be applied to calculate real shear rate and viscosity ${\dot{\gamma }}_{die\;}$ and $\eta_{die}$.

(5) $${\dot{\gamma }}_{die}=\frac{3m+1}{4m}\;\;{\dot{\gamma }}_{app}\;\;\;;\;\;\;\eta_{die}=\frac{4m}{3m+1}\;\;\eta_{app}\;;\;\mathrm{with}\;m-1=\frac{\partial \;\log\left(\eta \right)}{\partial \log\left(\dot{\gamma }\right)}$$

Usually, the parameter m is obtained by measuring several values of ${\dot{\gamma }}_{app}$ and $\;\eta_{app}$ and calculating the slope of the resulting line by plotting $log(\eta_{app})=f(log({\dot{\gamma }}_{app}$)). However, in the present study, it is impossible to obtain more than one point of the curve because measuring another pressure value means changing the process and thus changing the material itself. A first regression is then performed considering a Carreau-Yasuda model (Equation (6)) passing through this single point; it is thus considered that all degraded PE still follow a Carreau-Yasuda law. m and a parameters are set as the ones of the raw HDPE XRT70 (m = 0.058 and a = 0.248). They have been determined by a frequency sweep with the rheometer and a fitting with the Carreau-Yasuda model. The Cox-Merz law was assumed to compare the actual shear viscosity and the complex viscosity measured by oscillatory rheometry tests. According to the literature [35], the viscosity at high shear rates does not depend on molecular weight. The viscosity curves of different molecular weight polyethylene samples converge then on an identical power-law behaviour. Making this assumption, λ and $\eta_{0}$ have been determined for each sample through classical regression methods, and the equation is thus completely described.

(6) $$\eta \left(\dot{\gamma },\;T\right)=\eta_{0}{\cdot (1+{\left(\lambda \times \dot{\gamma }\right)}^{a})}^{\frac{m-1}{a}}$$

m and a = dimensionless indexes;

λ [s] = characteristic time;

$\eta_{0}$ [Pa·s] = zero shear viscosity.

The m parameter can then be calculated with the resulting curve, which enables the calculation of real shear rate ${\dot{\gamma }}_{die}\;$ and viscosity $\eta_{die}$ in the die thanks to Rabinowitsch correction. Finally, the real viscosity curve passing through this point is estimated by a second regression with a Carreau-Yasuda model in the same way as the first regression.

It is then possible to link the average molecular weight *M_w_* to *η*_0_ considering Equation (7). Often estimated at 3.4 for entangled polymers, the α exponent has been fixed at 3.6 in this study to be homogeneous with previous calculations and other HDPE studies [36,37,38] (the results being similar with the two values). The *K* constant can be determined from the values of *η*_0_ and *M_w_* of the reference HDPE.

(7) $$\eta_{0}=K\times M_{w}^{\alpha }$$

## 3. Modelling and Machine Learning

### 3.1. Simulation

Ludovic^®^ software (SC-Consultants, Saint-Etienne, France) is a well-known twin-screw extrusion simulation software. Due to its computing speed, ease of use and flexibility, it has proven its reliability in many different fields and applications. Initially developed for starch extrusion, it is now developed and used for plastics compounding and the pharmaceutical, cosmetics, food, and construction industries. This broad use makes it an interesting choice for this study and for comparison with new approaches such as machine learning.

Its efficiency is due to many simplifications and hypotheses and its adaptability to each situation. First, the melting process is considered instantaneous, but the user can also implement a melting model. Then, specific geometries are used depending on the element, allowing the flow calculation in only one dimension. Elements are divided into several sections in which the fluid is considered as Newtonian and isothermal. Specific viscosities depending on shear rate and temperature are however defined and chosen by the user. Dedicated articles were published for a complete description of the method [39].

Here, the software was configured to match screw profile, extruder, die and temperature profile with the experiments. Transfer coefficients were fixed at 50 W·m^−2^·K^−2^ for the die and 350 W·m^−2^·K^−2^ for the barrel, corresponding to similar and previous simulations [31,40].

Concerning the viscosity of the polyethylene, several options are available: Choosing a rheological model between Power Law and Carreau-Yasuda and indicating the corresponding parameters, implementing a new model, or entering a set of points (SoP) corresponding to rheological measurements. It is also possible to couple the viscosity with some simulation results, such as reaction rate (requires entering a description of the kinetics), total residence time, cumulated strain or total dissipated energy. Nevertheless, it requires knowing the relation between these parameters and viscosity. In our case, polyethylene viscosity evolves during the extrusion due to the degradation under high temperature. Berzin et al. [41] developed a method for coupling the starch viscosity variation with the SME on Ludovic^®^ (SC-Consultants, Saint-Etienne, France) However, here, the dependence between degradation and the process parameters is unknown, and the objective is to use the simulation classically.

HDPE viscosity has been defined as following a Carreau-Yasuda model, in which parameters have been determined from rheological measurements on the raw polyethylenes.

Concerning UHMWPE, it appears that the available rheological measurement methods underestimate the viscosity. Several hypotheses and simplifications had then to be made in order to approximate it for our simulation.

As a first simplification, the elastic modulus (*G′*) has been considered as constant and equal to its rubbery plateau value $G_{N}^{0}$, which can be calculated the following way:

(8) $$G_{N}^{0}=\frac{\rho R\;T}{M_{e}}$$

where ρ  is the density of the polymer estimated at 930 kg/m^3^ according to the supplier, R is the perfect gas constant, T is the reference temperature (*T* = 190 °C) and $M_{e}$ is the molecular weight between entanglements (1.25 kg/mol for polyethylene).

Then, as the loss modulus is significantly lower than the value of the elastic modulus, its contribution to viscosity calculation has been neglected.

Finally, the Cox-Merz hypothesis has been made, allowing to assimilate the actual viscosity η and shear rates $\dot{\gamma }\;$ of the extruder to the complex viscosity $\eta^{*}$ and angular frequency  ω, thus obtaining the following approximation:

(9) $$\eta \left(\dot{\gamma }\right)=\eta^{*}\left(\omega \right)=\frac{G\prime \left(\omega \right)}{\omega }=\frac{G_{N}^{0}}{\omega }$$

All the polymer characteristics used have been summarised in Table 3.

### 3.2. Machine-Learning

Since a data-driven model is only fed by data, the more data there is, the more accurate the prediction will be. While today, in the era of “big data”, one of the main concerns is to classify this huge amount of data successfully, this study is in the opposite situation dealing with the low data limit imposed by the number of extrusions and the available hardware. The dataset used in our study includes four types of inputs (HDPE or UHMWPE, flow rate, screw rotation speed and maximal imposed temperature), and only around 27 data, each corresponding to an extrusion configuration. To find a law linking the outputs to these inputs, it is consequently necessary to use algorithms able to perform with few data. As regression methods are well adapted to this case, Support Vector Machine Regression (SVR) and Sparsed Proper Generalized Decomposition (sPGD), two regression methods, have been tested for those data. Their specific modes of operation are described hereafter.

The process is as follows: the dataset is first randomly divided into training and test groups. Then, the training inputs and outputs are implemented in the algorithm, which, depending on the method, will “learn” from these data, creating a model linking the inputs to the outputs. The model is then tested with the remaining data, and the outputs predicted by it are compared to the real ones, allowing qualifying its accuracy.

#### 3.2.1. Support Vector Machine Regression—SVR

**Figure 5:**
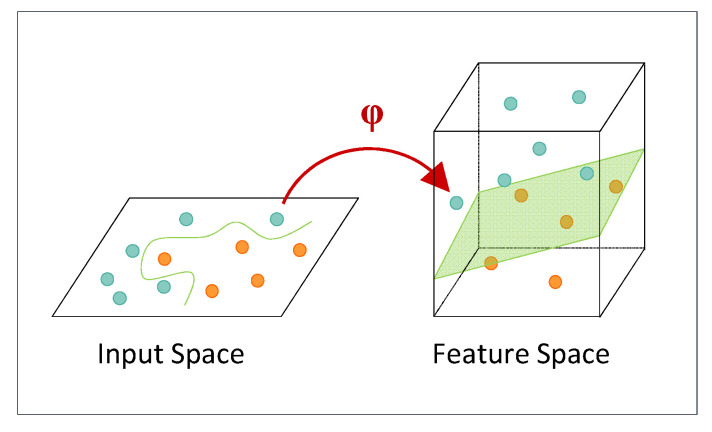
Scheme of SVM principle.

SVR is a derivate of the Support Machine Vector—SVM—classification method. It is a classical method known for its effectiveness in high dimensional spaces and is widely described in the literature, such as Smola et al. in 2004 [42]. As with all classification methods, SVM aims to find the equation of the limit between two classes. As it can be tricky or impossible to find this limit in the original space of the data, the strategy here is to represent the data in a higher-dimensional space where the equation of the limit would be more simple. This principle is represented in Figure 5. This transformation is carried out via a transformation function called “kernel” (noted *φ* in the figure). This kernel is defined by the user, depending on the system and its complexity. Here the “RBF” kernel was chosen as it is adapted to nonlinear systems.

The decision surface defining the different class areas is defined according to two parameters *C* and *ε* defined by the user. ε represents the maximum error between the decision surface and the experimental points, and *C* characterizes the smoothness of this surface. To be more specific, a high value of *C* would make the decision surface fit exactly all the experimental points but can lead to overfitting. It would hardly represent reality and would not fit new points that are not part of the training points. On the contrary, a low value of *C* would smooth the decision surface, enabling more errors and bringing more realism. This principle can also be used for regression purposes, and this is how the SVR method used here works. Then, the decision surface does not represent a border between classes but a hyper-surface approximating the points, predicting numerical values for new testing points.

#### 3.2.2. Sparsed Proper Generalized Decomposition—sPGD

This regression method has been developed and fully described by Ibáñez et al. [43]. To give an idea of the principle of this method, let us consider an output *y* that depends on two input parameters *x*_1_ and *x*_2_. The simplest regression method, particularly in the case of few experimental data, is a linear regression such as described by Equation (10).

(10) $$y\left(x_{1},x_{2}\right)=a+b\times x_{1}+c\times x_{2}$$

where three experimental data, i.e., values of ($y,x_{1},x_{2}$), are necessary to find *a*, *b* and *c* parameters of Equation (10).

However, the dependence between the inputs and outputs is often nonlinear, and calculating richer regressions would necessitate more data. The principle of sPGD is considering instead of (10) the following equation:

(11) $$y\left(x_{1},x_{2}\right)=X_{1}\left(x_{1}\right)\times X_{2}\left(x_{2}\right)$$

The dependence relation between the output *y* and the inputs $x_{1}$ and $x_{2}$ would thus be a product of functions depending each on $x_{1}$ and $x_{2}$. To determine these functions without needing more data, the following method is applied. First, $X_{1}\left(x_{1}\right)$ is fixed and $X_{2}\left(x_{2}\right)$ is estimated thanks to the available data. Only two data are then necessary to have a linear function in each coordinate and then a bilinear regression with only three data (some regularisation being needed to avoid unphysical behaviours). However, a more significant amount of data would lead to richer approximations. Then, $X_{1}\left(x_{1}\right)$ can be estimated by fixing $X_{2}\left(x_{2}\right)$ to its just calculated value using the same data. With this method, three experimental data could lead to a quadratic dependence instead of a linear one with Equation (10).

#### 3.2.3. Stochastic Methods

Classically, the way of estimating the equation matching a maximum with data is to apply the classical least-squares procedure. However, another method called “stochastic” based on statistics is possible.

Let consider a set of two variables $x_{i}$ and $y_{i}$ that we attempt to describe by a linear function $y_{i}=y_{i}\left(x_{i}\right)=ax_{i}+b$. The classical lest squares procedure consists of minimizing S.

(12) $$S=\frac{1}{2}\underset{i}{\sum }{\left(y_{i}-f\left(x_{i}\right)\right)}^{2}=\frac{1}{2}\underset{i}{\sum }{\left(y_{i}-\left(ax_{i}+b\right)\right)}^{2}$$

The number of samples is n (i=1..n).

The linear regression applied at each data point reads:

(13) $$b+ax_{1}=y_{1}\;\ldots \;b+ax_{n}=y_{n}$$

whose matric form reads

(14) $$\left(\begin{array}{cc} x_{1} & 1\\ \ldots & \ldots \\ x_{n} & 1 \end{array}\right)\left(\begin{array}{c} a\\ b \end{array}\right)=\left(\begin{array}{c} y_{1}\\ \ldots \\ y_{n} \end{array}\right)$$

or using a more compact form

(15) $$X\left(\begin{array}{c} a\\ b \end{array}\right)=Y$$

The least-squares procedure used for solving the just overdetermined system consists of pre-multiplying the previous system by $X^{T}$ that results in

(16) $$X^{T}X\left(\begin{array}{c} a\\ b \end{array}\right)=X^{T}Y$$

or more explicitly

(17) $$\left(\begin{array}{cc} \sum_{i}x_{i}^{2} & \sum_{i}x_{i}\\ \sum_{i}x_{i} & n \end{array}\right)\left(\begin{array}{c} a\\ b \end{array}\right)=\left(\begin{array}{c} \sum_{i}x_{i}y_{i}\\ \sum_{i}y_{i} \end{array}\right)$$

It is then possible to prove that the solution of this system results

(18) $$a=\frac{\mathrm{Cov}\left(x,y\right)}{\mathrm{Var}\left(x\right)}\;b=\bar{y}-a\bar{x}$$

with $\bar{y}\;$ and $\bar{x}$ the mean value in the sample of variables *y* and *x*, respectively.

We suppose now that for each input $x_{i}$ the response $y_{i}\;$ follow a Gaussian distribution with a standard deviation $\sigma_{i}$. Let denotes by $N_{\sigma }\left(.\right)$ the Gaussian distribution around the zero value with standard deviation σ. The least-squares procedure previously described is here modified. The idea consists in saying that each $y_{i}$ must follow a normal distribution centred at $ax_{i}+b$ and with a standard deviation $\sigma_{i}$.

The optimization problem results then in the maximization of the following sum

(19) $$S=\frac{1}{2}\underset{i}{\sum }N_{\sigma_{i}}\left(y_{i}-f\left(x_{i}\right)\right)=\frac{1}{2}\underset{i}{\sum }N_{\sigma_{i}}\left(y_{i}-\left(ax_{i}+b\right)\right)$$

To get back on a classical optimization problem expressed in terms of minimization, we just have to add a negative sign on the sum. The resolution cannot be made analytically, as was done in the case of the least-squares’ procedure. We can however use a descent scheme.

Assuming that the standard deviation does not depend on *x*, one could look for the statistical regression with a minimum standard deviation. For that, the most straightforward procedure consists of, for a tentative *a* and *b* coefficients of the regression, compute the standard deviation of the sampling, and then apply the previous rationale for computing the regression, that is, for updating *a* and *b* coefficients and iterate until reaching convergence.

The procedure described above is easily generalized to handle multiparametric regressions with richer nonlinear regressions. This method was tried on 10 data corresponding only to HDPE extruded at 190 °C and coupled to SVR previously described methodology.

## 4. Results and Discussion

### 4.1. Comparison of Estimated and Measured Viscosities

**Figure 6:**
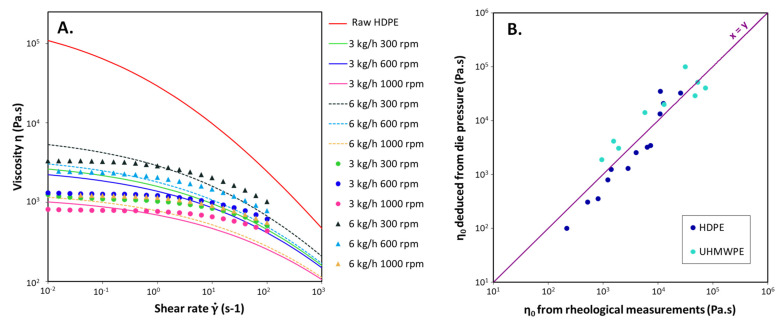
(**A**) Representation of calculated (lines) and measured (dots) viscosity curves for some HDPE extrusions at 390 °C for the indicated flow rates and screw rotation speeds; (**B**) Zero shear viscosities deduced from die pressure compared to the one obtained from rheological measurements.

The viscosities of the extruded materials deduced from the pressure measured at the die were compared to the rheometer measurements. The viscosity curves obtained from die pressure measurements are compared to data calculated from rheometer experiments in Figure 6A only for the samples resulting from HDPE 390 °C extrusion. Figure 6B compares the zero shear viscosities obtained for all samples with the two different methods. A Carreau-Yasuda model was thus used to fit the experimental viscosity curves, which validate the use of such a model for the degraded PE. With this type of representation, the closer the points on the *x* = *y* line are, the closer the values of the two methods for the same sample are. The samples from extrusions performed at different temperatures or with the UHMWPE give similar results. The results are fully detailed in Table A3 of the Appendix A.

Despite the assumptions made, the estimated viscosity curves are close to the rheometer experiments. However, the region of the Newtonian plateau at low frequency seems to be more pronounced for the rheometer data, leading to a higher value of the parameter a of the Carreau-Yasuda law. But the interest of this method is to avoid offline characterizations. Given the lack of information to model the curve correctly, it seems unlikely that better results can be obtained. Besides, the comparison of zero-shear viscosities gives satisfactory results and proves that this method can rapidly approximate the final viscosity of extruded materials.

### 4.2. Molecular Weight Distribution

**Figure 7:**
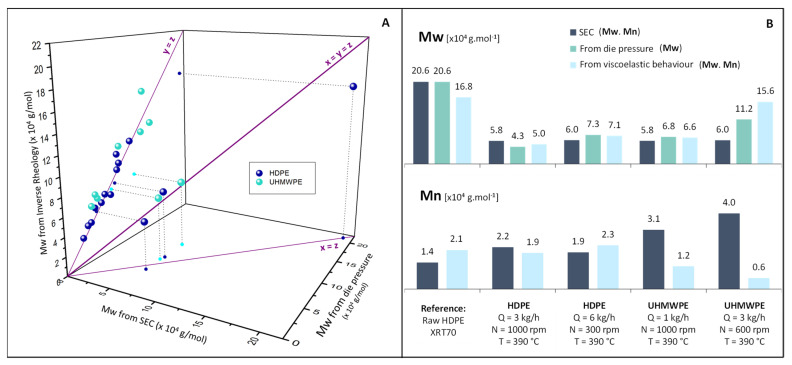
(**A**) Comparison of weight average molecular weight (*Mw*) of degraded polyethylenes obtained from the estimated zero shear rate viscosity versus the one obtained from viscoelastic behaviour measurements. (**B**) Comparison of weight average (*Mw*) and number average (*Mn*) molecular weight values obtained with different specified methodologies.

Figure 7A shows the comparison of the values of weight average molecular mass (*Mw*) obtained with the three methods previously described, i.e., (i) viscoelastic behaviour measurements, (ii) from die pressure and (iii) from size exclusion chromatography (SEC). Only the five samples analyzed by SEC are in the 3D figure, the other ones being in the die pressure versus viscoelastic behaviour plane. Figure 7B compares *Mw* values obtained by the three techniques and *Mn* values obtained by SEC and viscoelastic measurements. The results are fully detailed in Table A3 of the Appendix A. It appears that despite the simplifications they induce, the three methodologies give similar results for the determination of *Mw* for all samples, with a factor of less than 2 between values in most cases. Thus, it appears that, concerning the determination of *Mw*, the viscoelastic measurement method has few advantages over the die pressure method, which does not require offline characterizations. However, its advantages actually lie in the fact that this technique allows obtaining the complete distribution of molecular weights, unlike the die pressure method, which only allows obtaining the *Mw*.

**Figure 8:**
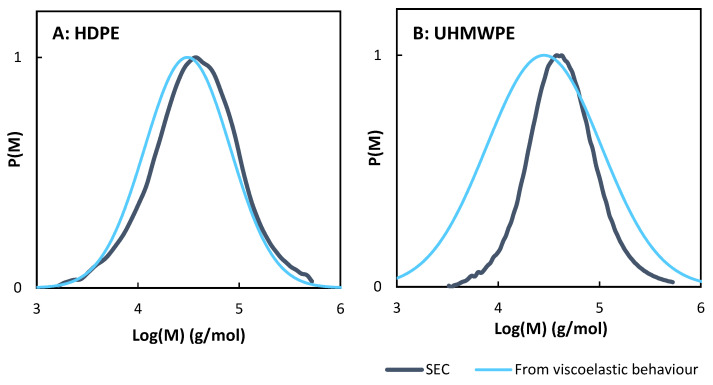
Molecular weight distribution obtained by SEC and viscoelastic measurements for (**A**) HDPE extruded at 390 °C, 3 kg/h and 1000 rpm and (**B**) UHMWPE extruded at 390 °C, 1 kg/h and 1000 rpm.

The *Mn* values obtained are compared to SEC values in Figure 7B. Figure 8 compares complete molecular weight distributions obtained by SEC and calculated from viscoelastic measurements for an HDPE and a UHMWPE. These figures point out that concerning HDPE samples, the distributions calculated are close to the ones obtained by SEC, which is the more precise methodology. It is consequently possible for this type of sample to determine the molecular weight distribution only from frequency sweep experiments, avoiding using SEC, which is a more complex and less accessible process involving the use of hot CMR solvents. However, for UHMWPE samples, *Mn* values are not as close to the SEC values. It can be due to structural differences with HDPE samples, inducing different parameters for the reptation model (relaxation time exponent, entanglement and reptation molecular weights, etc.).

### 4.3. Ludovic^®^ Simulation

**Figure 9:**
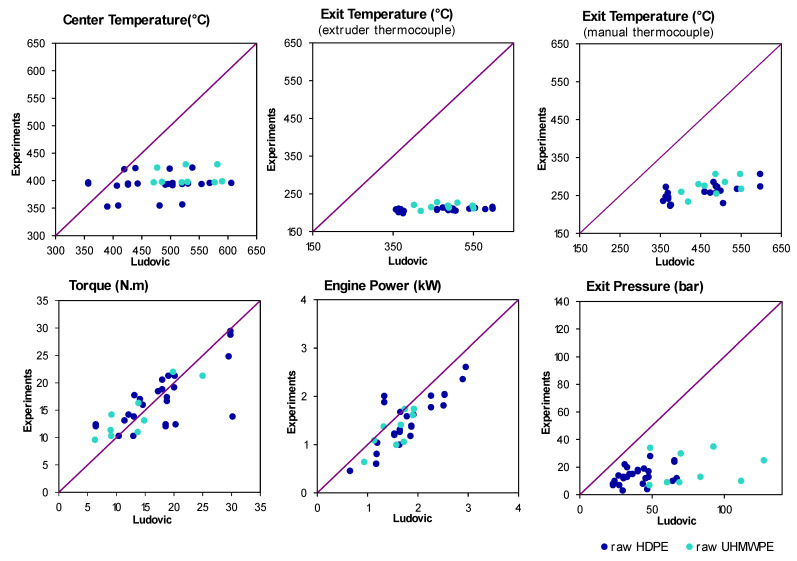
Comparison of Ludovic^®^ Simulation versus Experimental data for in-line measured parameters.

Figure 9 compares the values obtained with Ludovic^®^ simulation (*x*-axis) to the experimental measures (*y*-axis) for different process parameters. This representation involves that the closer the values are to the *x* = *y* line, the closer the predictions of Ludovic^®^ are to the measures. These results are fully detailed in Table A2 of the Appendix A.

The die temperatures measured by the extruder thermocouple are all around 200 °C, while the output temperatures measured with a manual thermocouple are much higher and more scattered. It is often a problem with extruder thermocouples which, being placed on the walls of the die, are influenced by its temperature and do not measure the actual melt temperature.

Concerning the simulation results, the first thing to notice is that all temperatures seem to be overestimated by the software, which matches the fact that polyethylene degradation is not considered. The actual viscosity decreases along with the screws and causes less self-heating than what could be expected without degradation. The temperature is, in fact, closer to the one imposed by the extruder.

This viscosity error also causes an overvaluation of the pressure in the die, more accentuated for UHMWPE because of its high viscosity. Concerning the torque and the engine power, and in the case of HDPE, the experimental values match pretty well with the simulation. It can be surprising considering the error between simulated and experimental viscosities caused by degradation. However, as viscosity decreases along the extruder, we can think that the torque value is mainly ruled by the most viscous part, which is the raw polyethylene present in the first screw elements and not yet degraded. The torque is then ruled by the viscosity of raw polyethylene, which is the one implemented in Ludovic^®^ (SC-Consultants, Saint-Etienne, France) The ability of machine learning algorithms to make better predictions than classic simulation is studied in what follows.

### 4.4. Data-Based Modelling

#### 4.4.1. Modelling of In-Line Measures with Machine-Learning Methods

This part presents the results obtained by Machine-Learning (ML) method on in-line measured parameters, which correspond to the parameters measured directly during the extrusion without needing additional experiments.

**Figure 10:**
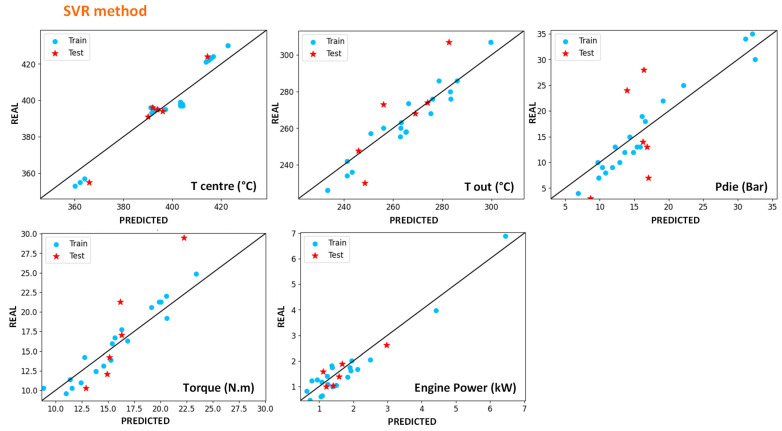
Results of the outputs predicted by the SVR method versus the experimental ones. The blue dots correspond to training data, and the red stars correspond to test data for HDPE and UHMWPE indiscriminately.

**Figure 11:**
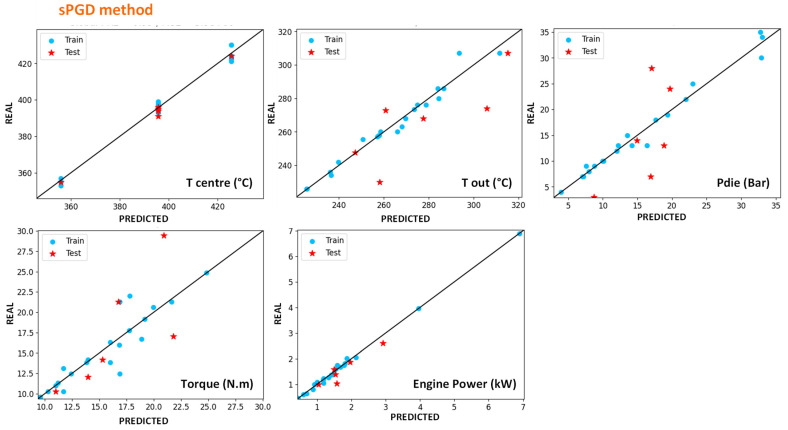
Results of the outputs predicted by the sPGD method versus the experimental ones. The blue dots correspond to training data, and the red stars correspond to test data for HDPE and UHMWPE indiscriminately.

Figure 10 and Figure 11 show the results obtained for centre and exit temperatures (manual thermocouple), torque, engine power and exit pressure for SVR and sPGD methods, respectively. To obtain these results, the model obtained after training with SVR regression has been applied on inputs and these figures represent the resulting outputs compared with the measured ones. Blue dots correspond to the data used for training and constructing the model, whereas the red star ones represent data that are new for the model as they have not been used for the training. Regarding these results, it appears that both methodologies give acceptable results as the dots are well distributed along the *x* = *y* line and relatively close to it.

In order to have more precise comparison tools, R^2^ scores were calculated on the results and presented in Table 4. The closer the score is to 1, the closer the model is to measures. Whereas obtaining a good score for training data is accessible, obtaining it for both training and test data is trickier. This table shows that both methods give acceptable errors but that the sPGD can be more precise for most parameters, particularly for the die pressure. On the contrary, exit temperature is a little bit more precisely modelled with SVR.

Finally, either of these methods gives better results than the classical Ludovic^®^ (SC-Consultants, Saint-Etienne, France) software model and good predictions without the need to understand the physical phenomena involved in the extrusion process. However, if this last point appears to be an advantage in favour of this method, it should be noted that one should be wary of it because the algorithm can model data that are false in the absolute. For example, the measured exit and centre temperature are very probably underestimated. Both algorithms, however, succeed to predict them, which proves that there is a logic between input parameters and these values. Nevertheless, they do not prevent eventual systematic errors in the measurements.

#### 4.4.2. Modelling of Viscosity and Molecular Weight

One of the main interesting aspects of data-based simulations is that there is no need to understand the physics behind the measurements to obtain predictive models of these results. Consequently, it is a less time and power-consuming way to obtain predictions for any measurements, as long as the correct inputs are given. ML methods can also succeed in predicting values depending on unknown phenomena. However, they work as black boxes and cannot help to understand these phenomena.

**Figure 12:**
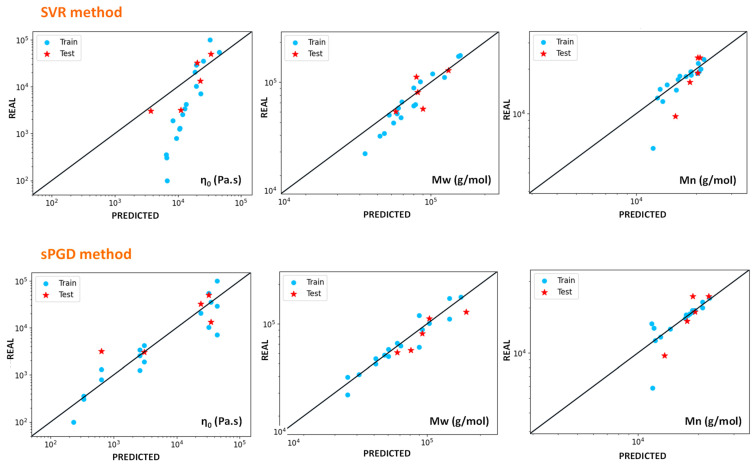
Results of zero-shear viscosity and weight and number average molecular weights predicted by SVR and sPGD methods versus the experimental ones. The blue dots correspond to training data, and the red stars correspond to test data for HDPE and UHMWPE indiscriminately.

For example, in this work, understanding the degradation mechanisms sufficiently to predict the final molecular weights of the material seems out of reach. On the contrary, predicting these values with ML methods seems entirely feasible. Figure 12 presents the results obtained with SVR and sPGD methods when predicting zero-shear viscosity *η*_0_ and weight and number average molecular weights *Mw* and *Mn*. Table 5 presents the R^2^ scores obtained, indicating the precision of these methods.

Both methods successfully model *Mw* values with reasonably high precision, but *Mn* values predictions are less accurate. *Mw* and *Mn* values implemented in the software are deduced from the viscoelastic behaviours of the melt samples. It has previously been shown that this method is more accurate for predicting *Mw* than *Mn*. This fact can explain the significant error noticeable for some of the data. Concerning the viscosity, the results obtained with the SVR method are pretty bad, and the predicted values seems shifted from the real ones. The cause of this phenomenon is unclear since the algorithm succeeds to predict *Mw* which, as seen previously, can directly be related to viscosity. sPGD algorithm presents similar R^2^ scores for viscosity, but less shifted values, which shows that the choice of the regression method is crucial.

#### 4.4.3. Stochastic Models

**Figure 13:**
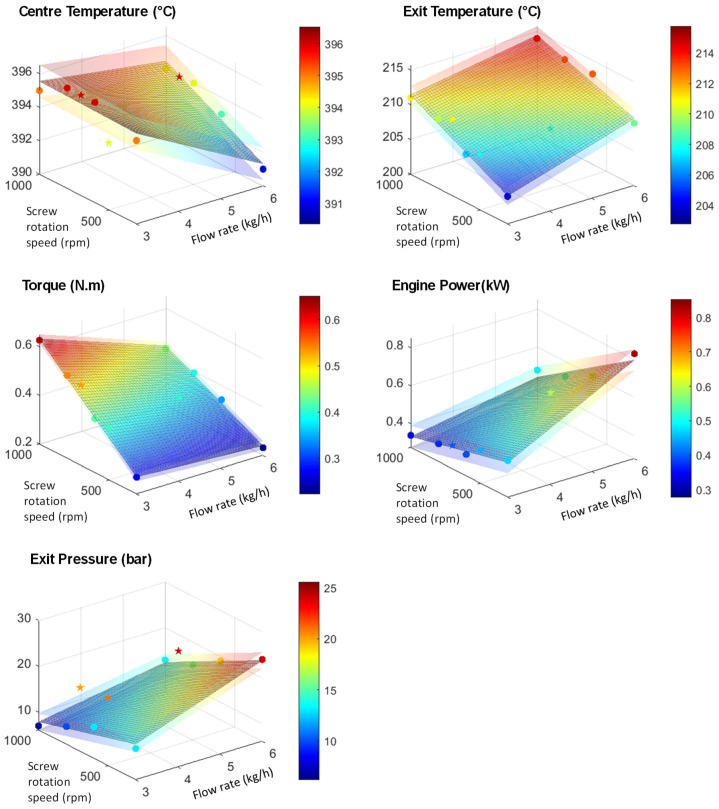
Results obtained with the stochastic method coupled to SVR. The dots correspond to training values, and the stars correspond to test ones.

One problem with data-driven models is that their accuracy depends on the accuracy of the data, which necessarily includes inaccuracies due to measurement techniques. Stochastic models allow considering probability curves instead of points as data, thus smoothing out these inaccuracies and generally simplifying the model. The SVR method coupled with the stochastic approach was tested on ten data corresponding to HDPE extruded at T_max_ = 390 °C. The results are shown in Figure 13.

As there are only two inputs for these data (Screw rotation speed and Flow rate), the results can be plotted in 3D graphs. The middle surface corresponds to the predictions, and the translucent ones correspond to the superior and inferior acceptation boundaries. Despite the limited amount of data, the method gave satisfactory results. Therefore, this method is promising and needs to be tested with the different polymers and temperatures as inputs and other outputs such as viscosities or molecular weights to see if the stochastic approach can make an improvement.

## 5. Conclusions

HDPE and UHMWPE were degraded by twin-screw extrusion under different high temperatures (320 < *T* °C < 420) and for different process conditions (flow rate and screw rotation speed), leading to numerous different extrusion configurations. Several parameters were measured for each configuration, creating a dataset with four different inputs, five outputs, and thirty-eight data.

The shear viscosity curves of the extruded materials were estimated from the measured die pressure and temperature. Their comparison with frequency sweep measurements showed that despite the numerous simplifications, the results were accurate. This fact shows that this method can be used to rapidly obtain an approximation of the final zero-shear viscosity of extruded materials.

Two methods were tested to estimate the molecular weight of extruded polyethylene. One was based on the viscoelastic behaviour of the material, and the other was deduced from die pressures and temperatures. The results showed that the average molecular weight *Mw* values were similar for both methods and similar to those obtained by SEC for the five samples tested. The method determining *Mw* only from measured die pressure and temperature thus seems more advantageous because it does not involve offline characterizations. However, this method is not sufficient to obtain the complete molecular weight distribution. In contrast, the other method based on viscoelastic measurements determined the complete molecular weight distribution. The results were good for HDPE but with some inaccuracies for UHMWPE samples. Although, as with the SEC, it requires offline characterizations, it is faster and is an interesting alternative.

The Ludovic^®^ (SC-Consultants, Saint-Etienne, France) twin-screw extrusion simulation software was used as a classical model of the extrusion experiments. Since the degradation mechanisms occurring in the extruder are unknown, the simulation was performed considering the viscosities of the raw materials, which led to overestimated pressures and temperatures. Consequently, SVR and sPGD Machine-Learning methods were applied to the dataset and succeeded in modelling the extrusions’ torque, engine power, die pressure, and die and centre temperatures. They also gave good results for the predictions of *Mw*. *Mn* has also been successfully predicted but with more inaccuracies, probably caused by its method of determination. Besides, whereas the SVR method gave inaccurate results for zero-shear viscosity modelling, sPGD’s results were more acceptable. Finally, stochastic methods were tested on ten of the data giving promising results.

Machine-Learning seems to be a valuable tool for extrusion simulation as it is possible to obtain quickly accurate models. However, it is essential to keep in mind that ML methods cannot be used as predictive tools and also that the accuracy of the results depends on the accuracy of the data. In perspective, it could be interesting to think about the scale-up of this process and about how machine-learning could be a helpful tool for this purpose. Few experiments on a larger scale could then be necessary to adapt the whole model to it.

## Figures and Tables

**Table 1:** Processing conditions used in this study.

Materials	T_max_ *	Flow Rate	Screw Rotation Speed
HDPE XRT70 TOTAL, (MFI = 0.7 g/10 min (190 °C, 5 kg))	350 °C 390 °C 420 °C	1 to 6 kg/h	300 to 1000 rpm
UHMWPE GUR 4130, Celanese, (MFI < 0.1 g/10 min (190 °C, 21.6 kg))	390 °C 420 °C	1 and 3 kg/h	300 to 1000 rpm

* T_max_ correspond to the maximal setpoint temperature cf. Figure 3.

**Table 2:** Parameters used for the molecular weight distribution calculation from inverse rheology method.

Symbol	Parameter	Value
*T*	Test Temperature	190 °C
*α*	Relaxation time exponent	3.6
$G_{N}^{0}$	Plateau modulus	2.3 × 10^6^ Pa
*E_a_*	Activation Energy	30 kJ/mol
*K_λ_*	Front Factor	2.5 × 10^−21^ s·(mol/g)^3.6^
*Me*	Entanglement Molecular weight	1250 g/mol
*Mr*	Reptation Molecular weight	2500 g/mol

**Table 3:** Thermal Properties and viscosity laws used in the simulation.

Thermal Properties	HDPE XRT70	UHMWPE GUR 4130
Heat Capacity [J kg^−1^ K^−1^]	1550	1840
Density [kg m^−3^]	947	930
Thermal Conductivity [W mK^−1^]	0.35	0.41
Melting Temperature [°C]	129	135
Melting enthalpy [kJ kg^−1^]	190	122
Viscosity Law	Carreau-Yasuda:	Power Law:
	$\eta \left(\dot{\gamma }\right)=\eta_{0}{\cdot (1+{\left(\lambda \times \dot{\gamma }\right)}^{a})}^{\frac{m-1}{a}}$	$\eta \left(\dot{\gamma }\right)=K\;\dot{\gamma }{\;}^{m-1}$
	$\eta_{0}\;$ = 2.5 × 10^6^ Pa·s λ = 0.33 s a = 0.25 m = 0.058 T_ref_ = 190 °C *E_a_* = 30 kJ·mol^−1^	m = 0 K = 2.86 × 10^6^ Pa·s T_ref_ = 190 °C *E_a_* = 30 kJ·mol^−1^

**Table 4:** R^2^ scores errors for SVR and sPGD methods.

R^2^ Error	Centre Temperature	Exit Temperature	Torque	Engine Power	Die Pressure
SVR train	0.93	0.93	0.91	0.93	0.92
SVR global	0.92	0.88	0.8	0.92	0.75
sPGD train	0.99	0.91	0.82	1	0.98
sPGD global	0.99	0.88	0.71	0.99	0.84

**Table 5:** R^2^ scores errors for the determination of *η*_0_, *Mw* and *Mn* with SVR and sPGD methods.

R^2^ Error	*η*_0_	*Mw*	*Mn*
SVR train	0.48	0.91	0.77
SVR global	0.50	0.86	0.70
sPGD train	0.49	0.90	0.77
sPGD global	0.50	0.82	0.75

## Data Availability

Data is contained within the article.

## Appendix A. Detail of Data’s

**Table A1:** In-line measurements for the different tested extrusion parameters.

Process Inputs				Extrusion in-Line Measurements				
PE Type	*Q* (kg/h)	N (rpm)	T_max_ (°C)	Torque (N·m)	Pe (Bar)	*Tc* (°C)	Engine Power (kW)	Te (°C)
HDPE	3	300	350	21.3	28	355	1.04	248
HDPE	3	600	350	17.7	22	357	1.68	273
HDPE	6	300	350	13.8	12	353	3.97	236
HDPE	6	600	350	12.4	12	355	6.89	258
HDPE	1	300	390	10.3	7	395	0.46	226
HDPE	3	300	390	16.7	13	395	0.81	242
HDPE	3	300	390	17.4	17	393	0.81	247
HDPE	3	500	390	16.0	15	394	1.23	260
HDPE	3	500	390	16.0	15	394	1.20	261
HDPE	3	600	390	13.8	12	395	1.31	263
HDPE	3	700	390	14.2	14	396	1.59	268
HDPE	3	1000	390	12.4	8	397	2.01	274
HDPE	5	600	390	20.6	18	396	2.01	276
HDPE	6	300	390	28.7	25	391	0.69	273
HDPE	6	600	390	21.3	19	393	1.02	286
HDPE	6	1000	390	17.0	20	396	0.38	307
HDPE	3	300	420	12.4	4	423	0.60	226
HDPE	3	600	420	10.3	3	424	1.00	230
HDPE	6	300	420	24.8	10	421	0.59	257
HDPE	6	600	420	19.2	8	422	1.81	258
UHMWPE	1	300	390	13.1	25	397	0.64	260
UHMWPE	1	600	390	11.4	13	398	1.09	276
UHMWPE	1	1000	390	9.6	9	399	1.38	286
UHMWPE	3	400	390	22.0	35	398	1.41	280
UHMWPE	3	600	390	16.3	30	397	1.74	307
UHMWPE	3	1000	390	14.2	34	397	1.74	307
UHMWPE	3	300	420	21.3	10	424	1.00	234
UHMWPE	3	600	420	11.0	9	430	1.06	256
UHMWPE	3	1000	420	10.3	7	430	1.61	268

**Table A2:** Ludovic^®^ results obtained for the different tested extrusion parameters.

Process Inputs				Ludovic^®^ Simulation				
Polymer	*Q* (kg/h)	N (rpm)	T_max_ (°C)	Torque (N·m)	Te (°C)	*Tc* (°C)	Pe (bar)	Engine Power (kW)
HDPE	3	300	350	19.0	363	409	49	1.19
HDPE	3	600	350	13.1	491	520	31	1.65
HDPE	6	300	350	30.2	357	390	67	1.90
HDPE	6	600	350	20.3	473	481	45	2.55
HDPE	1	300	390	10.4	376	443	27	0.65
HDPE	3	300	390	18.7	369	426	47	1.18
HDPE	3	500	390	14.6	459	496	34	1.53
HDPE	3	500	390	14.6	459	496	34	1.53
HDPE	3	600	390	13.0	499	530	30	1.64
HDPE	3	700	390	12.1	539	568	27	1.78
HDPE	3	800	390	11.4	579	606	24	1.91
HDPE	3	1000	390	6.4	597	356	23	1.33
HDPE	5	600	390	17.9	487	503	40	2.26
HDPE	6	300	390	29.8	363	407	65	1.87
HDPE	6	600	390	20.1	481	491	44	2.53
HDPE	6	800	390	17.2	553	554	36	2.89
HDPE	6	1000	390	14.1	597	520	32	2.95
HDPE	3	300	420	18.5	374	439	47	1.17
HDPE	3	600	420	12.9	505	538	30	1.63
HDPE	6	300	420	29.5	368	419	64	1.85
HDPE	6	600	420	20.0	488	498	43	2.51
UHMWPE	1	300	390	14.8	402	471	127	0.93
UHMWPE	1	600	390	9.0	459	530	83	1.13
UHMWPE	1	1000	390	6.3	510	590	60	1.32
UHMWPE	3	400	390	19.8	444	485	92	1.66
UHMWPE	3	600	390	13.8	486	519	70	1.74
UHMWPE	3	1000	390	9.2	547	577	49	1.92
UHMWPE	3	300	420	25.0	419	477	112	1.57
UHMWPE	3	600	420	13.7	488	527	69	1.72
UHMWPE	3	1000	420	9.1	550	581	48	1.90

**Table A3:** Viscosities and molecular weights for the different tested extrusion parameters.

Process Inputs				Zero Shear-Rate Viscosities		Molecular Weights (Inverse Rheology)			*Mw* (From Die Pressure)
Polymer	*Q* (kg/h)	N (rpm)	*T* (°C)	*η*_0_ from Die Pressure (Pa·s)	*η*_0_ Rheometer (Pa·s)	*Mw* (g/mol)	Mz (g/mol)	*Mn* (g/mol)	*Mw* (g/mol)
HDPE	3	300	350	2.6 × 10^4^	3.2 × 10^4^	1.1 × 10^5^	5.0 × 10^5^	2.4 × 10^4^	1.1 × 10^5^
HDPE	3	600	350	1.3 × 10^4^	2.1 × 10^4^	9.1 × 10^4^	4.3 × 10^5^	1.9 × 10^4^	8.6 × 10^4^
HDPE	6	300	350	1.1 × 10^4^	3.5 × 10^4^	1.0 × 10^5^	5.2 × 10^5^	1.9 × 10^4^	8.3 × 10^4^
HDPE	6	600	350	1.1 × 10^4^	1.3 × 10^4^	8.4 × 10^4^	3.7 × 10^5^	1.9 × 10^4^	8.2 × 10^4^
HDPE	3	300	390	6.4 × 10^3^	3.2 × 10^3^	6.0 × 10^4^	1.5 × 10^5^	2.4 × 10^4^	7.0 × 10^4^
HDPE	6	300	390	7.4 × 10^3^	3.4 × 10^3^	7.1 × 10^4^	2.2 × 10^5^	2.3 × 10^4^	7.3 × 10^4^
HDPE	6	600	390	4.0 × 10^3^	2.5 × 10^3^	6.4 × 10^4^	2.0 × 10^5^	2.0 × 10^4^	6.1 × 10^4^
HDPE	6	1000	390	1.4 × 10^3^	1.2 × 10^3^	5.4 × 10^4^	1.5 × 10^5^	1.9 × 10^4^	4.5 × 10^4^
HDPE	3	300	420	3.5 × 10^2^	9.0 × 10^1^	2.8 × 10^4^	5.9 × 10^4^	1.3 × 10^4^	3.0 × 10^4^
HDPE	3	600	420	2.2 × 10^2^	1.0 × 10^2^	2.9 × 10^4^	5.9 × 10^4^	1.4 × 10^4^	2.6 × 10^4^
HDPE	6	300	420	8.1 × 10^2^	3.5 × 10^2^	4.1 × 10^4^	9.3 × 10^4^	1.8 × 10^4^	3.8 × 10^4^
HDPE	6	600	420	5.2 × 10^2^	3.1 × 10^2^	3.9 × 10^4^	8.7 × 10^4^	1.8 × 10^4^	3.4 × 10^4^
UHMWPE	1	300	390	7.4 × 10^4^	5.0 × 10^4^	1.2 × 10^5^	1.4 × 10^6^	9.6 × 10^3^	1.4 × 10^5^
UHMWPE	1	600	390	1.3 × 10^4^	5.4 × 10^4^	1.1 × 10^5^	8.8 × 10^5^	1.3 × 10^4^	8.6 × 10^4^
UHMWPE	1	1000	390	5.8 × 10^3^	1.0 × 10^4^	6.6 × 10^4^	3.5 × 10^5^	1.2 × 10^4^	6.8 × 10^4^
UHMWPE	3	400	390	5.3 × 10^4^	7.0 × 10^3^	1.6 × 10^5^	1.5 × 10^6^	1.6 × 10^4^	1.3 × 10^5^
UHMWPE	3	600	390	3.1 × 10^4^	1.0 × 10^5^	1.6 × 10^5^	3.4 × 10^6^	5.8 × 10^3^	1.1 × 10^5^
UHMWPE	3	1000	390	4.8 × 10^4^	2.9 × 10^4^	1.2 × 10^5^	8.7 × 10^5^	1.5 × 10^4^	1.3 × 10^5^
UHMWPE	3	300	420	1.9 × 10^3^	3.1 × 10^3^	6.3 × 10^4^	2.4 × 10^5^	1.6 × 10^4^	4.9 × 10^4^
UHMWPE	3	600	420	1.5 × 10^3^	4.2 × 10^3^	6.8 × 10^4^	2.6 × 10^5^	1.8 × 10^4^	4.6 × 10^4^
UHMWPE	3	1000	420	9.4 × 10^2^	1.9 × 10^3^	5.8 × 10^4^	2.0 × 10^5^	1.7 × 10^4^	4.6 × 10^4^
